# Multigene profiles to guide the use of neoadjuvant chemotherapy for breast cancer: a Copenhagen Breast Cancer Genomics Study

**DOI:** 10.1038/s41523-023-00551-0

**Published:** 2023-05-31

**Authors:** M.-B. Jensen, C. B. Pedersen, M.-A. Misiakou, M.-L. M. Talman, L. Gibson, U. B. Tange, H. Kledal, I. Vejborg, N. Kroman, F. C. Nielsen, B. Ejlertsen, M. Rossing

**Affiliations:** 1 Danish Breast Cancer Cooperative Group, Rigshospitalet, Copenhagen University Hospital, Copenhagen, Denmark; 2 Center for Genomic Medicine, Rigshospitalet, Copenhagen University Hospital, Copenhagen, Denmark; 3 Section for Bioinformatics, DTU Health Technology, Technical University of Denmark, Kongens Lyngby, Denmark; 4 Department of Pathology, Rigshospitalet, Copenhagen University Hospital, Rigshospitalet, Copenhagen, Denmark; 5 Department of Clinical Oncology, Rigshospitalet, Copenhagen University Hospital, Copenhagen, Denmark; 6 Department of Breast Examinations, Copenhagen University Hospital, Herlev-Gentofte, Copenhagen, Denmark; 7 Department of Breast Surgery, Herlev-Gentofte Hospital, Copenhagen University Hospital, Copenhagen, Denmark; 8 Department of Clinical Medicine, University of Copenhagen, Copenhagen, Denmark

**Keywords:** Predictive markers, Breast cancer

## Abstract

Estrogen receptor (ER) and human epidermal growth factor 2 (HER2) expression guide the use of neoadjuvant chemotherapy (NACT) in patients with early breast cancer. We evaluate the independent predictive value of adding a multigene profile (CIT256 and PAM50) to immunohistochemical (IHC) profile regarding pathological complete response (pCR) and conversion of positive to negative axillary lymph node status. The cohort includes 458 patients who had genomic profiling performed as standard of care. Using logistic regression, higher pCR and node conversion rates among patients with Non-luminal subtypes are shown, and importantly the predictive value is independent of IHC profile. In patients with ER-positive and HER2-negative breast cancer an odds ratio of 9.78 (95% CI 2.60;36.8), *P* < 0.001 is found for pCR among CIT256 Non-luminal vs. Luminal subtypes. The results suggest a role for integrated use of up-front multigene subtyping for selection of a neoadjuvant approach in ER-positive HER2-negative breast cancer.

## Introduction

The use of neoadjuvant chemotherapy (NACT) has increased significantly among patients with operable breast cancer. Reducing the risk of potential morbidity by less invasive surgery due to downstaging or optimizing breast-contour preservation was the primary driving force^1,2^. Demonstration of a prognostic impact in achieving pathological complete response (pCR) further promoted the use of NACT, particularly for patients with a high probability of obtaining pCR, e.g., those with estrogen receptor (ER) negative or human epidermal growth factor receptor-2 (HER2) positive breast cancers^3^. Finally, the predictive ability to guide post-neoadjuvant systemic therapy further promoted the concept^4,5^.

Compared to other subtypes, the patients with ER-positive and HER2-negative (defined as patients with HER2 0, 1+, or 2+ and a low ratio) cancers (approximately 70% of the population) are considerably less likely to achieve pCR but have the same improved outcome following pCR^3,6^. ER-positive and HER2-negative disease is both clinically and biologically heterogeneous and several characteristics including tumor size, malignancy grade, Ki67, PR, and genomic profiles have been associated with the ability to achieve pCR^7–11^. However, evidence to support the use of multigene profiles or other factors to guide the decision of selecting NACT is insufficient in ER-positive and HER2-negative cancers, and the general view is that NACT should only be considered if adjuvant chemotherapy is recommended irrespective of surgical pathology data^1^. Intrinsic molecular subtyping has emerged as a predictor of breast cancer recurrence in ER-positive and HER2-negative cancers^12,13^. The 50‐gene molecular classifier (PAM50) reproduces the five originally proposed subclasses, and the vast majority of ER-positive, HER2-negative tumors are classified as Luminal A, Luminal B, or Normal-like. However, a small part will be classified as HER2‐enriched or Basal-like tumors^8^. Using integrative analysis of both genomic and transcriptomic data, six stable molecular subtypes (assigned CIT256 in the following) have more recently been derived, based on genomic rearrangement and expression of 256 genes^14,15^, with the additional subtype Luminal C (LumC) enabling the distinction and capture of subtypes in a heterogenic background. In this six-subtype scheme, the HER2-positive tumors primarily clustered into the molecular Apocrine (mApo) and LumC subtypes, with reference LumC samples having lower *ESR1* expression than those of the Normal-like (NormL), Luminal A (LumA), and Luminal B (LumB) subtypes. NormL, LumA, and LumB were composed of highly ER-positive tumors with Basal-like (BasL) and mApo being at the other end of the spectrum and inversely related to malignancy grade^14^.

In this study we evaluate the strength of multigene profiles from pretreatment core needle biopsies performed as a standard of care diagnostic pipeline, to predict pCR.

## Results

### Patient characteristics

**Fig. 1:**
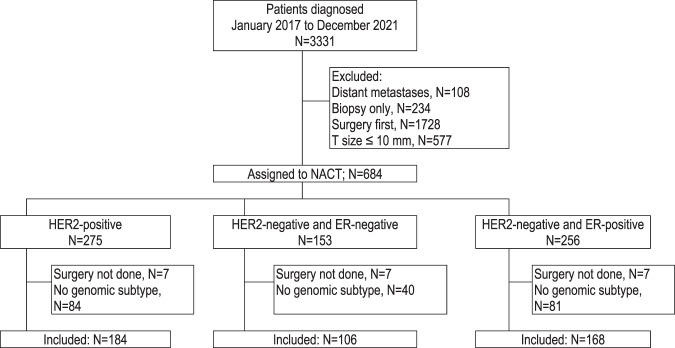
Flow diagram of patient cohort in CBCSG. ER Estrogen receptor, HER2 Human epidermal growth factor receptor-2.

Among the 3331 patients registered in CBCGS between January 2017 and December 2021, 684 were assigned to NACT while 108 had distant metastases, 234 not were eligible for surgery, 1728 were treated with surgery first, and 577 had a tumor ≤ 10 mm. A subtype was not available for 205 of the NACT patients, and 21 had no surgery, leaving 458 eligible for this study (Fig. 1).

### Multigene profiles

**Fig. 2:**
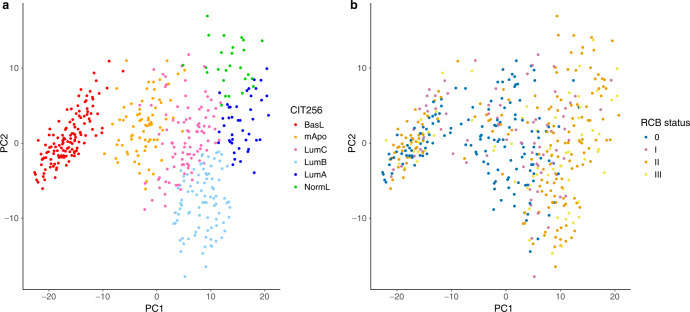
Principal component analysis of the 458 samples. **a** The distribution of the six subtypes based on the 375 probe sets of the CIT256 classifier; NormL (green), LumA (dark blue), LumB (light blue), LumC (pink), mApo (orange) and BasL (red). **b** The distribution according to RCB class; 0 (blue), I (pink), II (orange), and III (yellow). BasL Basal-like, mApo molecular Apocrine, LumA Luminal A, LumB Luminal B, LumC Luminal C, NormL Normal-like, PC Principal component, RCB Residual Cancer Burden.

The CIT256 scheme resulted in the following distribution of molecular subtypes: BasL 28%, mApo 16%, LumC 20%, LumB 20%, LumA 10%, and NormL 6% (Table 1, Fig. 2).

**Table 1:** Patient and tumor characteristics by genomic subtype CIT256.

Characteristics	Study population		Genomic subtype CIT256												
			BasL		mApo		LumC		LumB		LumA		NormL		
	*N*	(%)	*N*	(%)	*N*	(%)	*N*	(%)	*N*	(%)	*N*	(%)	*N*	(%)	*P*
	458		130	(28)	74	(16)	93	(20)	91	(20)	44	(10)	26	(6)	
Age															<0.001
<40 Years	84	(18)	45	(35)	10	(14)	12	(13)	13	(14)	1	(2)	3	(12)	
40–49	163	(36)	41	(32)	25	(34)	34	(37)	32	(35)	18	(41)	13	(50)	
50–59	112	(24)	26	(20)	16	(22)	29	(31)	23	(25)	11	(25)	7	(27)	
≥60	99	(22)	18	(14)	23	(31)	18	(19)	23	(25)	14	(32)	3	(12)	
Node status															0.002
Negative	264	(58)	88	(68)	33	(45)	49	(53)	46	(51)	27	(61)	21	(81)	
Positive	194	(42)	42	(32)	41	(55)	44	(47)	45	(49)	17	(39)	5	(19)	
Tumor size UL															0.09
≤20 mm	76	(16)	26	(20)	16	(22)	11	(12)	17	(19)	3	(7)	3	(12)	
21–50 mm	302	(66)	87	(67)	45	(61)	66	(71)	58	(64)	32	(73)	14	(54)	
>50 mm	75	(16)	14	(11)	13	(18)	15	(16)	15	(16)	9	(20)	9	(35)	
Unknown	5	(2)	3	(2)	0	(0)	1	(1)	1	(1)	0	(0)	0	(0)	
Histologic type															0.004
Ductal	409	(89)	113	(87)	62	(84)	86	(92)	88	(97)	38	(86)	22	(85)	
Lobular	14	(3)	0	(0)	3	(4)	2	(2)	1	(1)	4	(9)	4	(15)	
Other	35	(8)	17	(13)	9	(12)	5	(5)	2	(2)	2	(5)	0	(0)	
Malignancy grade															<0.001
Grade I	46	(10)	0	(0)	2	(3)	12	(13)	9	(10)	15	(34)	8	(31)	
Grade II	212	(46)	35	(27)	38	(51)	48	(52)	53	(58)	23	(52)	15	(58)	
Grade III	160	(35)	83	(64)	28	(38)	25	(27)	21	(23)	2	(5)	1	(4)	
Unknown	40	(9)	12	(9)	6	(8)	8	(9)	8	(9)	4	(9)	2	(8)	
ER status															<0.001
Positive^a^	291	(64)	29	(22)	20	(27)	83	(89)	91	(100)	44	(100)	24	(92)	
Negative	167	(36)	101	(78)	54	(73)	10	(11)	0	(0)	0	(0)	2	(8)	
HER2 status															<0.001
Negative	274	(60)	118	(91)	15	(20)	24	(26)	55	(60)	38	(86)	24	(92)	
Positive	184	(40)	12	(9)	59	(80)	69	(74)	36	(40)	6	(14)	2	(8)	
IHC profile															<0.001
ER- HER2-	106	(23)	91	(70)	13	(18)	0	(0)	0	(0)	0	(0)	2	(8)	
ER- HER2+	61	(13)	10	(8)	41	(55)	10	(11)	0	(0)	0	(0)	0	(0)	
ER + HER2−	168	(37)	27	(21)	2	(3)	24	(26)	55	(60)	38	(86)	22	(85)	
ER + HER2+	123	(27)	2	(2)	18	(24)	59	(63)	36	(40)	6	(14)	2	(8)	
PAM50															<0.001
Basal-like	160	(35)	130	(100)	16	(22)	8	(9)	6	(7)	0	(0)	0	(0)	
HER2-enriched	110	(24)	0	(0)	54	(73)	52	(56)	4	(4)	0	(0)	0	(0)	
Luminal A	66	(14)	0	(0)	0	(0)	9	(10)	15	(16)	28	(64)	14	(54)	
Luminal B	85	(19)	0	(0)	0	(0)	13	(13)	63	(69)	10	(23)	0	(0)	
Normal-like	37	(8)	0	(0)	4	(5)	12	(13)	3	(3)	6	(14)	12	(46)	
BRCA mutation															<0.001
No	332	(72)	96	(74)	58	(78)	64	(69)	60	(66)	31	(70)	23	(88)	
BRCA1	28	(6)	20	(15)	3	(4)	2	(2)	3	(3)	0	(0)	0	(0)	
BRCA2	17	(4)	6	(5)	2	(3)	1	(1)	6	(6)	2	(5)	0	(0)	
Not tested	81	(18)	8	(6)	11	(15)	26	(28)	22	(24)	11	(25)	3	(12)	
Chemotherapy															0.15
3CE + 3TAX	240	(52)	80	(33)	30	13)	47	(29)	47	(20)	24	(10)	12	(5)	
4CE + 4TAX	131	(29)	35	(27)	24	(18)	24	(18)	25	(19)	14	(11)	9	(7)	
≤6 cycles^b^	56	(12)	7	(13)	15	(27)	13	(23)	11	(20)	5	(9)	5	(9)	
7-8 cycles^c^	31	(7)	8	(26)	5	(16)	9	(29)	8	(26)	1	(3)	0	(0)	
Anti-HER2 therapy (*N* = 184 HER2-positive)															
T (trastuzumab)	13	(7)	1	(8)	1	(8)	7	(54)	3	(23)	1	(8)	0	(0)	
T and P (pertuzumab)	5	(3)	1	(20)	1	(20)	3	(60)	0	(0)	0	(0)	0	(0)	
TP → T/T-DM1^d^	166	(90)	10	(6)	57	(34)	59	(36)	33	(20)	5	(3)	2	(1)	

^a^23 patients with ER 1–9%, 10 were HER2-positive (1 BasL, 7 mApo and 2 LumC) and 13 HER2-negative (12 BasL and 1 mApo).

^b^Includes patients with TAX alone (*N* = 3), with CE alone (*N* = 3), and with combinations other than 3 + 3 (*N* = 26 less than 6).

^c^Includes patients with TAX alone (*N* = 1) and with combinations other than 4 + 4 (*N* = 17 less than 8).

^d^TP preoperatively, T/T-DM1 post-surgery. *N* = 93 HER2-positive and no pCR; hereof 20 treated with T-DM1 post-surgery.

*P*-value refer to χ^2^-test or Fisher’s exact test.

*UL* Ultrasound localization, *IHC* Immunohistochemistry, *ER+* Estrogen receptor positive, *ER*− ER-negative, *HER2*− Human epidermal growth factor receptor-2-negative, *HER2+* HER2-positive, *CE* Cyclophosphamide and Epirubicin, *TAX* paclitaxel (or docetaxel), *T* Trastuzumab, *P* Pertuzumab, *T-DM1* Trastuzumab emtansine, *BasL* Basal-like, *mApo* molecular Apocrine, *LumA* Luminal A, *LumB* Luminal B, *LumC* Luminal C, *NormL* Normal-like.

**Fig. 3:**
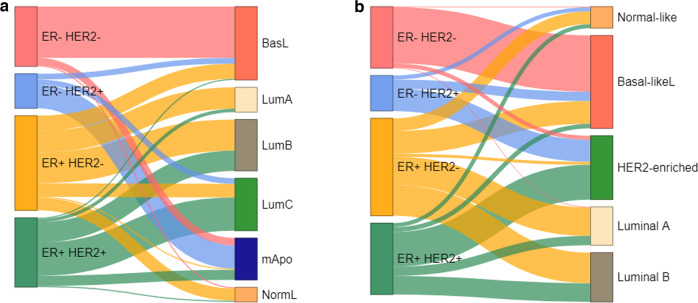
Sankey plot between IHC subtypes and multigene profiles. **a** CIT256 profile, **b** PAM50 profile. ER Estrogen receptor, HER2 Human epidermal growth factor receptor-2, ER+ ER-positive, ER− ER-negative, HER2+ Her2-positive, HER2− HER2−negative, BasL Basal-like, mApo molecular Apocrine, LumA Luminal A, LumB Luminal B, LumC Luminal C, NormL Normal-like.

A significant association with the CIT256 was seen for all parameters, except chemotherapy and tumor size, presented in Table 1. The association of CIT256 and ER status revealed no patients with ER-negative tumors in the LumA or LumB subtypes and only two patients in the NormL subtype, and similarly, few patients in the Luminal PAM50 subtypes (Fig. 3, Supplementary Table S1). Among 168 patients with ER-positive HER2-negative tumors 32% present with a Non-luminal (BasL, mApo, LumC) subtype (Table 1). From the relation between CIT256 and PAM50 very few patients are seen in discordant (Non-luminal vs. Luminal) subgroups, except for the LumC subtype (Table 1).

### pCR rate according to IHC profile and multigene subtypes

RCB class was correlated to ER and HER2 status as well as multigene subtypes with higher response rate in subgroups with ER-negative vs. ER-positive, HER2-positive vs. HER2-negative and Non-luminal vs. Luminal genomic subtypes (Supplementary Table S2, Fig. 2). Number of patients and pCR rate according to IHC profile, CIT256 and PAM50 multigene subtypes are presented in Supplementary Table 3.

**Fig. 4:**
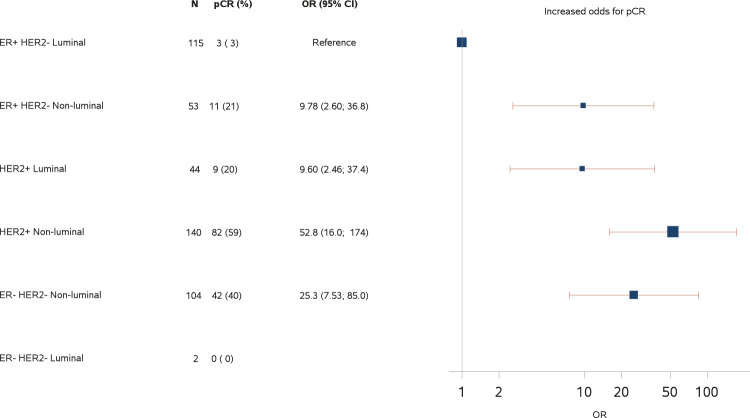
Forest plot from logistic regression model of pathological complete response according to IHC profile and CIT256 profile combined. A supplementary analysis excluding ER 1–9% from ER+ resulted in a point estimate of 9.33 for ER + HER2- Non-luminal. The boxes represent the weight of data in terms of sample size for each subgroup and the lines represent the 95% confidence interval of the estimated odds ratio. ER Estrogen receptor, HER2 Human epidermal growth factor receptor-2, ER+ ER-positive, ER− ER-negative, HER2+ Her2-positive, HER2− HER2-negative, Luminal Luminal A, Luminal B Normal-like, Non-luminal Basal-like, HER2-enriched, pCR Pathological complete response (Residual Cancer Burden 0), OR odds ratio, IHC Immunohistochemistry.

The corresponding unadjusted estimates for odds ratio are presented and all three profiles show highly statistical significance (*P* < 0.001), with low pCR rate for the ER-positive, HER2-negative group and a low pCR rate for the Luminal multigene profile for both CIT256 and PAM50. The independent predictive effect of the IHC profile and multigene subtype was confirmed in the multivariable regression model, where both parameters remained highly statistically significant (*P* < 0.001, Supplementary Table 4). The predictive accuracy for the univariable models were 0.721 (95%CI 0.677;0.765), 0.699 (95%CI 0.663;0.734), and 0.692 (95%CI 0.652;0.732) for the IHC -, the CIT256 - and the PAM50 profile, respectively, with a significantly better predictive performance (*P* < 0.001) in the combined IHC and multigene profile; 0.780 (95%CI 0.740;0.821) for IHC and CIT256 and 0.776 (95%CI 0.734;0.817) for IHC and PAM50 combined (Supplementary Table 5). The test for heterogeneity revealed no significance, implying that the effect of the multigene profile remained within each IHC subgroup (and vice versa). This is also evident from the odds ratio estimates for the combined (IHC profile and multigene profile) subgroups presented in Fig. 4 and Supplementary Fig. 1 with a very low pCR rate of 3% in patients with concordant IHC (ER-positive, HER2-negative) and multigene (Luminal) estrogen positive tumors. A more detailed overview of the combination of IHC profile, multigene subtype and pCR status is provided in Supplementary Table S1 and odds ratio estimates for all subgroups in the IHC and multigene profiles in Supplementary Table S3, where also the relation between Luminal A and Luminal B is presented.

Focusing on the ER-positive, HER2-negative IHC subgroup, there was a clear distinction in the pCR rate for patients grouped in the CIT256 Non-luminal vs. Luminal subtypes, with an estimated odds ratio of 9.78 (95% confidence interval (CI) 2.60;36.8), *P* < 0.001 (Fig. 4), and a similar odds ratio estimate for PAM50 Non-luminal vs. Luminal 8.82 (95%CI 2.60;29.0), *P* < 0.001 (Supplementary Fig. 1).

### *BRCA* mutation

Pathogenic *BRCA1* and *BRCA2* germline mutations were identified in 28 and 17 patients, respectively, 332 had no *BRCA1/2* mutation while 81 were not tested. Twenty-six (58%) of the patients with a *BRCA* mutation were classified in the CIT256 BasL subtype (Table 1). Type of surgery following chemotherapy for the subgroup of patients with no *BRCA* mutation detected, is presented in Supplementary Table S6. In total 54% of the patients had breast conserving surgery (BCS).

### Axillary status following NACT

Conversion of axillary status is presented in Supplementary Table S7 for the ER-positive, HER2-negative IHC subgroup. Among patients classified with a positive nodal status at diagnosis based on fine needle aspiration, a very low number of the patients in the Luminal subgroup have a negative nodal status following NACT with 3 (7%) in the CIT256 profile and 5 (10%) in the PAM50 Luminal group. These figures are higher for the Non-luminal subgroup with 9 (36%) and 7 (32%) for CIT256 and PAM50, respectively, both with statistical significance (*P* = 0.002 and *P* = 0.03) comparing Non-luminal with Luminal. This is in line with the pattern seen for pCR.

## Discussion

Our study confirmed the association of estrogen receptor and HER2 expression with pCR as previously suggested by the pooled analysis of NACT trials published in 2014^3^. Furthermore, we demonstrated that the multigene profiles CIT256 and PAM50 add significant predictive information for pCR and RCB class in patients with a tumor size >10 mm as well as for conversion of cytologically verified positive axillary nodes. This is especially important for women with ER-positive and HER2-negative breast cancer where less than 10% overall obtained a pCR but by using multigene profiles they could be separated into two distinct subgroups; those with luminal subtypes having only a 3% chance of achieving pCR compared with a >20% probability of pCR among those with non-luminal subtypes. In the patients with ER-positive, HER2-negative status and a Luminal CIT256 multigene profile, only 3 patients (7%) were converted to a node-negative status following NACT compared to 36% among those with a Non-luminal profile. For patients with clinically positive nodes, downstaging may obviate extensive axillary surgery and thereby reduce the risk of lymphedema, pain, and dysesthesias^16^.

Gene expression signatures provide prognostic information beyond standard clinicopathological factors. Large clinical trials have utilized multigene subtyping to improve the treatment choice for patients with luminal breast cancer, more specifically by defining which patients should be offered chemotherapy, and which patients can be adequately treated with endocrine therapy alone^17,18^. The results of three large clinical trials with focus on precision medicine in women with hormone receptor positive and HER2-negative breast cancer showed that postmenopausal women with high-risk features defined by standard clinical factors (tumor size, nodal status, malignancy grade), but at low risk of recurrence according to genomic assays, could safely be allocated to endocrine therapy alone, whereas a benefit of chemotherapy in premenopausal women was shown consistently^17,19^. Additionally, the publications by Farrugia et al. and Bhargava et al. have demonstrated the strong predictive power of Magee Equation 3, which is based on ER, PR, HER2, and Ki-67 in prediction of pCR among ER-positive and HER2-negative breast cancer patients treated with NACT^11,20^. Furthermore, secondary analyses investigated the integrated use of clinical risk factors and information from the gene scores on both prognosis and the predictive effect of adding chemotherapy to endocrine treatment. Clinical risk stratification added prognostic information beyond the genomic scores and thereby confirmed that the integrated use of both provide a more accurate estimation of risk of recurrence, whereas the clinical risk factors were not complementary to the predictive information of the assay^18^.

The heterogeneity in pCR according to PAM50 subtypes confirms the observations made by Ohara and colleagues who, by using PAM50, classified 124 patients with ER-positive breast cancer as Luminal A (52), Luminal B (32), HER2-Enriched (24), or Basal-like (16). The pCR rate was 4.8% in patients with luminal compared to 20.0% in patients with Non-luminal cancers^9^. However, the Ohara study included 31 HER2-positive patients which even in the absence of HER2 targeting treatment may explain the difference. Parker et al. included 133 patients with both ER-positive and -negative status, as well as HER2-negative and -positive status, and both Basal-like, HER2-enriched and Luminal B, showed odds ratios of 2–3 as compared to Luminal A subtype^8^, whereas the pCR rate was not reported. In a study including ER-positive and HER2-negative breast cancers only a few patients were classified by PAM50 as non-luminal and primarily compared pCR rates according to Luminal A vs. Luminal B^21^ based on very sparse data. In a recent abstract Ma et al. presented data on 93 ER-positive and HER2-negative breast cancer patients who participated in the ALTERNATE trial which suggested a higher pCR rate of 16.7% in patients with non-luminal cancers compared to 4.0% in patients with Luminal A or Luminal B subtypes^22^.

Furthermore, other assays such as BluePrint and MammaPrint were investigated^23,24^ with similar outcomes, but a poor concordance of only 59% comparing BluePrint and PAM50 was shown^25^. Several smaller studies have shown a significant association between the Recurrence Score (RS) and pCR following NACT^26–30^ while a few have not^31,32^, and a significant association with pCR after NACT was shown for a high RS (OR 4.87, 95% CI 2.01, 1.82) in 898 ER-positive and HER2-negative breast cancer patients^10^. Response-predictive subtyping were investigated in the I-SPY2 neoadjuvant platform trial including 987 patients, and also reported low pCR rates in the patients with ER-positive, HER2-negative tumors. Treatment allocation based on subtyping schemas showed increased pCR rates, and the improved patient selection was highest in group of patients with hormone-receptor positive disease^33^.

The strengths of the present study include the prospective identification and multigene testing of eligible patients within a single institution. Furthermore, both treatment and outcome of the diagnostic procedure were prospectively registered in an independent clinical database of a cooperative group, allowing a high level of completeness. The concordance between the findings of CIT256 and PAM50 makes the results applicable for open-source platforms. Although both dichotomized CIT256- and PAM50-based subtyping status was significantly associated with the rate of pCR, we did observe slightly different odds ratios for the two subtyping schemes. Perhaps this can be explained by the fact that assignment of patients to the LumC subtype of the CIT256 scheme provides important information for NACT response. This subtype is considered part of the good-response Non-luminal class, but 36% of the patients assigned to this subtype are assigned to the Luminal subtypes for the PAM50 scheme.

The current study has some potential limitations. Although the study cohort is of a reasonable size, subgrouping according to both IHC profile and multigene profile resulted in small sample sizes for each of the subtypes. In order to increase the statistical power and avoid multiple testing of multigene subtypes, two subsets were constructed according to *ESR1* profiling, proliferation and luminal type. In patients with HER2-positive breast cancer the pCR rate observed in patients with a luminal subtype may not be sufficiently low to clinically impact the decision to offer NACT. For patients with ER-negative and HER2-negative breast cancers, multigene subtyping is also unlikely to impact the decision to offer NACT as all but two patients were assigned to a non-luminal subtype. It is important to note that the patients of this study were assigned to NACT without consideration of the multigene subtype and it can prove difficult to ensure availability of the test result before initiation of treatment. However, with the implementation of molecular subtyping in the clinical workflow, a multigene subtyping result can be at hand within seven days^34^. In conclusion, this study demonstrates that molecular subtyping may be used as guidance in the selection of a neoadjuvant approach when chemotherapy is indicated for patients with ER-positive and HER2-negative breast cancers.

## Materials and methods

### Study population

The Copenhagen Breast Cancer Genomics Study (CBCGS) prospectively enrolled patients age 18 or older who were diagnosed with invasive breast cancer at Copenhagen University Hospital, Rigshospitalet between January 2014 and December 2021. Detailed information on diagnosis, genomic profiling, treatment and follow-up was registered in the clinical Danish Breast Cancer Group (DBCG) database. Eligible for the present study were patients free of distant metastasis, had a tumor size >10 mm, and were recommended NACT. Molecular subtyping was part of the routine diagnostic work-up. NACT consisted of 3-weekly epirubicin and cyclophosphamide for three or four cycles followed by weekly paclitaxel for 9–12 cycles. HER2-positive patients received 3 or 4 cycles of trastuzumab and pertuzumab in conjunction with the taxane and continued HER2 targeted therapy postoperatively. Following NACT, surgery was according to national guidelines; patients with a clinically negative axilla at time of diagnosis were recommended sentinel lymph node biopsy, and axillary lymph node dissection was recommended for all patients with lymph node metastases. Residual Cancer Burden (RCB) was calculated using the Residual Cancer Burden Calculator from MD Anderson Cancer Center^35^.

This register-based study was conducted with approval of the Danish Data Protection Agency (jr. no.: 2012-58-0004, 30-1504 I-Suite 03845) and the Danish Breast Cancer Group (jr. no.: DBCG-2015-14). All participants provided written, informed consent before clinical and biomarker study data was entered into the DBCG database. As the study did not include any contact with patients nor additional use of biological material, the need to obtain a re-consent from participants for this sub-analysis, was waived by the Ethical Committee of the Capital Region of Denmark.

### Assessment of ER, HER2, BRCA status, and RNA extraction

Standard histopathological diagnosis was performed from formalin-fixed, paraffin-embedded tissue blocks, on breast core needle biopsies, according to the WHO-classification recommendations^36^, including immunohistochemical staining for ER and HER2, at time of diagnosis. These analyses were repeated on the surgical specimens following NACT in case of non-pCR, using tissue microarray technique, with two cores of 2 mm from the invasive front of each tumor. Areas suitable for these cores, were marked on hematoxylin and eosin stained slides by an experienced breast pathologist^37^. In case of very small tumor size, whole mount slides replaced the TMA technique. A cutoff point of ≥1% for ER-positive tumors was used. Scoring of HER2 was performed following the ASCO/CAP guidelines^38^. Pretreatment core biopsies for gene expression profiling were collected in RNAlater stabilization solution (Thermo Fisher Scientific, Waltham, MA, USA), and total RNA was isolated, and used as the starting material for gene expression quantification with the Human Genome U133 Plus 2.0 Array (Afymetrix, Santa Clara, CA, USA), as previously described^39^.

Mutation screening was done using the breast cancer-predisposing gene panel, as previously described^40^. *BRCA1* and *BRCA2* likely pathogenic (class 4) and pathogenic (class 5) variants were considered as positive status.

### NGS library preparation

For RNA sequencing, 200 ng input RNA was used for preparing RNA sequencing libraries, which was done using TruSeq Stranded Total RNA Library Prep Kit (Illumina) following manufacturer’s instructions. Briefly, RNA was denatured and ribosomal RNA (rRNA) was removed using Ribominus Gold (Illumina). After cleanup, RNA was fragmented, and 1st and 2nd strand synthesis performed. Finally, libraries were adaptor ligated and amplified. AMPure XP beads were used for cleaning up the prepared sequencing libraries and quantification was done using a Qubit fluorometer (Termo Fisher Scientific, Waltham, MA, USA). Sequencing libraries were paired-end sequenced (2 × 125 bp) on the Illumina HiSeq2500 platform.

### Data preprocessing and molecular subtyping scheme

For each microarray sample, the raw intensity.CEL file was preprocessed together with 30 existing breast cancer samples from Rigshospitalet by quantile normalization, and probe summaries were extracted via robust multi-array average (RMA) using the *affy* package in R v. 4.0.0^41^. Subsequently, ComBat^42^ from the *sva* package^43^ was applied for batch correction of 12 of the reference samples and the sample of interest together with the CIT256^15^ core set, as presented previously^34^. Sample origin was used as batch and initially predicted CIT256 subtypes (determined using the *citbcmst* R-package) acted as covariates.

For RNA-seq, the reads from each sample were used as input for two independent computational pipelines. In the first pipeline, samples were processed according to the method presented by Pedersen et al.^44^. Briefly, raw fastq files were aligned to the probe target sequences from the Affymetrix Human Genome U133 Plus 2.0 Array using kallisto v. 0.44.0, and the resulting transcript per million values (TPM) per probe set were processed in R v. 4.0.0 together with a set of 18 existing breast cancer samples from Rigshospitalet representative of each molecular subtype. TPM values were quantile-normalized to the row mean values from the CIT256^15^ core set using the *preprocessCore* package^45^, and the two data sets were batch-corrected using ComBat^42^ from the sva package^43^, considering the two sets as distinct batches. This was followed by initial prediction of CIT256 subtypes, which were used as covariates in a second batch correction with ComBat with the same two batches as above.

In the second pipeline, fastq files were mapped to the human GRCh37.p13 reference genome using STAR^46^ aligner v. 2.7.2b and reads overlapping features were quantified using Subread package’s featureCounts v. 1.6.2^47^. Subsequently values for each sample were merged together with the same set of 18 existing breast cancer samples mentioned above and normalized by size factors determined by the median ratio of gene counts relative to geometric mean per gene using *DeSeq2* R package^48^.

For the CIT256 scheme, one of six subtypes (BasL, mApo, LumA, LumB, LumC, NormL) was assigned to each sample by the CIT256 tool using a distance-to-centroid approach relying on expression of 375 probe sets^15^. For the PAM50 molecular subtyping scheme, for RNA-seq samples, log2-transformed normalized expression values were used as input for the original predictor developed by Parker et al.^8^. The classifier calculates Spearman’s rank correlation between each sample and each subtype centroid for the 50 genes of interest and assigns the class (Luminal A, Luminal B, HER2-enriched, Basal-like, Normal-like) of the most highly correlated centroid to each sample. For microarray normalized expression values the *genefu* R package was used for assigning a PAM50 subtype based on the Pearson correlation to the PAM50 centroids.

### Statistical analysis

The primary endpoint was pathological complete response (pCR) corresponding to RCB class 0 and secondary endpoints were RCB class and pCR in the axilla. Associations of genomic CIT256 subtype and RCB class with patient and tumor characteristics were analyzed using χ^2^ and Fisher’s exact tests, excluding unknowns. Univariate logistic regression was performed to assess odds ratios for pCR according to genomic subtype and immunohistochemical (IHC) profile (ER-status and HER2-status combined). Genomic subtyping was analyzed in separate subtypes and grouped based on *ESR1* profiling, proliferation and “luminal type”. The latter was defined based on genomic subtypes: For CIT256, high *ESR1* profile/low proliferation subtypes (LumA, LumB, NormL) were referred to as Luminal and low *ESR1* profile/high proliferation subtypes (LumC, mApo, BasL) were termed Non-luminal. Since LumC is a boundary subtype showing the largest genomic similarity with the mAPO-subtype, samples assigned with LumC were also termed as Non-luminal. For PAM50, we considered Luminal (Luminal A, Luminal B, Normal-like) vs. Non-luminal (Basal-like, HER2-enriched). Further, IHC profile and genomic subtype combined were analyzed. Multivariable logistic regression was used to assess the independent effect of genomic subtype and IHC profile by including both, applying the Wald test. Predictive performance for the logistic regression models is presented by the c-statistic. All tests were two-sided and a *P* value < 0.05 was considered statistically significant. No adjustment for multiple testing was done. All analyses were performed using SAS Enterprise Guide version 7.15, SAS Institute Inc., Cary and R version 4.1.2.

### Reporting summary

Further information on research design is available in the Nature Research Reporting Summary linked to this article.

## Supplementary information

Reporting Summary

Supplementary material

## Data Availability

The clinical data that support the findings of this study are available from the corresponding author upon reasonable request and with permission from the host institution but restrictions apply to the availability. Microarray data are available on GEO (GSE231629) and RNA-seq data is available on Zenodo (10.5281/zenodo.7898803).
