# Supplementary material for: Multigene profiles to guide the use of neoadjuvant chemotherapy for breast cancer: a Copenhagen Breast Cancer Genomics Study
Source: NPJ Breast Cancer. 2023 May 31;9:47. doi: 10.1038/s41523-023-00551-0 (PMC10232408; doi:10.1038/s41523-023-00551-0)
Supplement: Supplementary file 1 — Reporting Summary [file 41523_2023_551_MOESM1_ESM.pdf]

nature portfolio

Corresponding author(s): Maj-Britt Jensen

Last updated by author(s): May 15, 2023

Reporting Summary

Nature Portfolio wishes to improve the reproducibility of the work that we publish. This form provides structure for consistency and transparency in reporting. For further information on Nature Portfolio policies, see our Editorial Policies and the Editorial Policy Checklist.

Statistics

For all statistical analyses, confirm that the following items are present in the figure legend, table legend, main text, or Methods section.

n/a	Confirmed	
☐	☒	The exact sample size (*n*) for each experimental group/condition, given as a discrete number and unit of measurement
☐	☒	A statement on whether measurements were taken from distinct samples or whether the same sample was measured repeatedly
☐	☒	The statistical test(s) used AND whether they are one- or two-sided<br>*Only common tests should be described solely by name; describe more complex techniques in the Methods section.*
☐	☒	A description of all covariates tested
☐	☒	A description of any assumptions or corrections, such as tests of normality and adjustment for multiple comparisons
☐	☒	A full description of the statistical parameters including central tendency (e.g. means) or other basic estimates (e.g. regression coefficient) AND variation (e.g. standard deviation) or associated estimates of uncertainty (e.g. confidence intervals)
☐	☒	For null hypothesis testing, the test statistic (e.g. *F*, *t*, *r*) with confidence intervals, effect sizes, degrees of freedom and *P* value noted<br>*Give P values as exact values whenever suitable.*
☒	☐	For Bayesian analysis, information on the choice of priors and Markov chain Monte Carlo settings
☒	☐	For hierarchical and complex designs, identification of the appropriate level for tests and full reporting of outcomes
☒	☐	Estimates of effect sizes (e.g. Cohen's *d*, Pearson's *r*), indicating how they were calculated

Our web collection on statistics for biologists contains articles on many of the points above.

Software and code

Policy information about availability of computer code

Data collection	Data were collected in the Danish Breast Cancer Group clinical database, using customised data entry forms programmed in php and storing data in an Oracle database.
Data analysis	SAS Enterprise Guide version 7.15, SAS Institute Inc., Cary and R version 4.0.0 and version 4.1.2

For manuscripts utilizing custom algorithms or software that are central to the research but not yet described in published literature, software must be made available to editors and reviewers. We strongly encourage code deposition in a community repository (e.g. GitHub). See the Nature Portfolio guidelines for submitting code & software for further information.

Data

Policy information about availability of data

All manuscripts must include a data availability statement. This statement should provide the following information, where applicable:

- Accession codes, unique identifiers, or web links for publicly available datasets
- A description of any restrictions on data availability
- For clinical datasets or third party data, please ensure that the statement adheres to our policy

The clinical data that support the findings of this study are available from the corresponding author upon reasonable request and with permission from the host institution but restrictions apply to the availability. Microarray data is available on GEO (GSE231629; https://www.ncbi.nlm.nih.gov/geo/query/acc.cgi?acc=GSE231629) and RNA-seq data is available on Zenodo (10.5281/zenodo.7898803;

Human research participants

Policy information about studies involving human research participants and Sex and Gender in Research.

Reporting on sex and gender	N=458 female breast cancer patients age 18 or older were included. Only female patients were included in the DBCG database for the specific inclusion period. In Denmark, sex and age are given from the specific Civil Registration Number assigned to each citizen and used for identification in the database.
Population characteristics	Female breast cancer patients who were diagnosed with invasive breast cancer at Copenhagen University Hospital, Rigshospitalet between January 2014 and December 2021. Eligible for the present study were patients free of distant metastasis, had a tumor size >10 mm, were treated with neoadjuvant chemotherapy, and had surgery following NACT.
Recruitment	Patients with the above mentioned characteristics, and where molecular subtyping as part of the routine diagnostic was performed were eligible for recruitment. A flow-diagram has been included to outline the selection of patients.
Ethics oversight	The study was approved by the Danish Data Protection Agency (jr. no.: 2012-58-0004, 30-1504 I-Suite 03845) and the Danish Breast Cancer Group (jr. no.: DBCG-2015-14). All participants provided written, informed consent before clinical and biomarker study data was registered and the need to obtain a re-consent from participants for this sub-analysis, was waived by the Ethical Committee of the Capital Region of Denmark.

Note that full information on the approval of the study protocol must also be provided in the manuscript.

Field-specific reporting

Please select the one below that is the best fit for your research. If you are not sure, read the appropriate sections before making your selection.

☒ Life sciences ☐ Behavioural & social sciences ☐ Ecological, evolutionary & environmental sciences

For a reference copy of the document with all sections, see nature.com/documents/nr-reporting-summary-flat.pdf

Life sciences study design

All studies must disclose on these points even when the disclosure is negative.

Sample size	No sample-size calculation was done. The study included all patients eligible according to the inclusion criteria. The study provided highly statistically significant results indicationg sufficient sample size.
Data exclusions	Among patients assigned to NACT, patients with no surgery or no genomic subtype established were not included. These exclusion criteria were pre-established to ensure the genomic subtype and also the primary outcome; pathologic complete response.
Replication	No replication were performed. Results on IHC profile confirms findings from other studies, and the results on the two different genomic profiles used in the study were very alike, which have been adressed in the manuscript.
Randomization	The study did not include experimental groups.
Blinding	Blinding was not performed. The treatment and outcome were part of routine diagnostic work-up and treatment.

Reporting for specific materials, systems and methods

We require information from authors about some types of materials, experimental systems and methods used in many studies. Here, indicate whether each material, system or method listed is relevant to your study. If you are not sure if a list item applies to your research, read the appropriate section before selecting a response.

Materials & experimental systems

n/a	Involved in the study	
☒	☐	Antibodies
☒	☐	Eukaryotic cell lines
☒	☐	Palaeontology and archaeology
☒	☐	Animals and other organisms
☐	☒	Clinical data
☒	☐	Dual use research of concern

Methods

n/a	Involved in the study	
☒	☐	ChIP-seq
☒	☐	Flow cytometry
☒	☐	MRI-based neuroimaging

Clinical data

Policy information about clinical studies

All manuscripts should comply with the ICMJE guidelines for publication of clinical research and a completed CONSORT checklist must be included with all submissions.

Clinical trial registration: NA except for Danish Data Protection Agency (jr. no.: 2012-58-0004, 30-1504 I-Suite 03845) .

Study protocol: The study is a cohort study based on prospectively collected data on routine diagnostic work-up and treatment.

Data collection: Patients were diagnosed in the time period January 2017 to December 2021. Data were collected prospectively in the Danish Breast Cancer Group clinical database.

Outcomes: The primary endpoint was pathological complete response (pCR) corresponding to RCB class 0 and secondary endpoints were RCB class and pCR in the axilla.
