# Supplementary material for: Multigene profiles to guide the use of neoadjuvant chemotherapy for breast cancer: a Copenhagen Breast Cancer Genomics Study
Source: NPJ Breast Cancer. 2023 May 31;9:47. doi: 10.1038/s41523-023-00551-0 (PMC10232408; doi:10.1038/s41523-023-00551-0)
Supplement: Supplementary file 2 — Supplementary material [file 41523_2023_551_MOESM2_ESM.pdf]

Supplementary material

Supplementary Tables 1-7

Supplementary Table 1. Number of patients and response according to IHC based subtypes combined with multigene profiles.

Supplementary Table 2. Residual cancer burden (RCB) class 0-III according to tumor characteristics.

Supplementary Table 3. Univariable logistic regression model analyses of pathological complete response according to IHC and multigene profiles.

Supplementary Table 4. Multivariable logistic regression models of pathological complete response according to IHC profile and multigene profiles.

Supplementary Table 5. True classification and model predicted classification from scoring of model with IHC and CIT256 profile and IHC and PAM50 profile.

Supplementary Table 6. Post-NACT surgery for patients with no *BRCA* mutation detected according to IHC biomarker and multigene profiles.

Supplementary Table 7. Axillary status before and after NACT for patients with ER-positive, HER2-negative breast cancer according to multigene profiles.

Supplementary Figure 1. Forest plot from logistic regression model of pathological complete response according to IHC profile and PAM50 profile combined.

Supplementary Table 1. Number of patients and response according to IHC based subtypes combined with multigene profiles.

	Genomic subtype CIT256																	
	BasL			mApo			LumC			LumB			LumA			NormL		
	N	pCR		*N*	pCR		*N*	pCR		*N*	pCR		*N*	pCR		*N*	pCR	
		N	(%)		*N*	(%)		*N*	(%)		*N*	(%)		*N*	(%)		*N*	(%)
IHC profile																		
ER- HER2-	91	37	(41)	13	5	(38)	0	0	(0)	0	0	(0)	0	0	(0)	2	0	(0)
ER- HER2+	10	4	(40)	41	25	(61)	10	6	(60)	0	0	(0)	0	0	(0)	0	0	(0)
ER+ HER2-	27	7	(26)	2	0	(0)	24	4	(17)	55	3	(5)	38	0	(0)	22	0	(0)
ER+ HER2+	2	2	(100)	18	15	(83)	59	30	(51)	36	7	(19)	6	1	(17)	2	1	(50)
HER2+[1]	12	6	(50)	59	40	(68)	69	36	(52)	36	7	(19)	6	1	(17)	2	1	(50)

	Genomic subtype PAM50														
	Basal-like			HER2-enriched			Luminal A			Luminal B			Normal-like		
	N	pCR		*N*	pCR		*N*	pCR		*N*	pCR		*N*	pCR	
		N	(%)		*N*	(%)		*N*	(%)		*N*	(%)		*N*	(%)
IHC profile															
ER- HER2-	97	39	(40)	7	3	(43)	1	0	(0)	0	0	(0)	1	0	(0)
ER- HER2+	16	6	(38)	38	23	(61)	0	0	(0)	0	0	(0)	7	6	(86
ER+ HER2-	39	7	(18)	5	3	(60)	49	0	(0)	53	3	(6)	22	1	(5)
ER+ HER2+	8	4	(50)	60	40	(67)	16	2	(13)	32	7	(22)	7	3	(43)
HER2+ [1]	24	10	(42)	98	63	(64)	16	2	(13)	32	7	(22)	14	9	(64)

[1]ER- HER2+ and ER+ HER2+ combined.
Abbreviations: BasL, Basal-like; mApo, molecular Apocrine; LumA, Luminal A; LumB, Luminal B; LumC, Luminal C; NormL, Normal-like; pCR, Pathological complete response (Residual Cancer Burden 0); IHC, Immunohistochemistry; ER+, Estrogen receptor positive; ER-, ER-negative; HER2-, Human epidermal growth factor receptor-2-negative; HER2+, HER2-positive.

Supplementary Table 2. Residual cancer burden (RCB) class 0-III according to tumor characteristics.

	Residual Cancer Burden								
	RCB 0		RCB I		RCB II		RCB III		
**Characteristics**	***N***	**(%)**	***N***	**(%)**	***N***	**(%)**	***N***	**(%)**	***P***
	147	(32)	73	(16)	172	(38)	66	(14)	
Histologic type									0.05
Ductal	125	(31)	66	(16)	157	(38)	61	(15)	
Lobular	3	(21)	2	(14)	5	(36)	4	(29)	
Other	19	(54)	5	(14)	10	(29)	1	(3)	
Malignancy grade									<.001
Grade I	3	(7)	11	(24)	24	(52)	8	(17)	
Grade II	65	(31)	33	(16)	75	(35)	39	(18)	
Grade III	63	(39)	28	(18)	54	(34)	15	(9)	
Unknown	16	(40)	1	(3)	19	(48)	4	(10)	
ER status									<.001
Positive	70	(24)	45	(15)	122	(42)	54	(19)	
Negative	77	(46)	28	(17)	50	(30)	12	(7)	
HER2 status									<.001
Normal	56	(20)	35	(13)	123	(45)	60	(22)	
Positive	91	(49)	38	(21)	49	(27)	6	(3)	
IHC profile									<.001
ER- HER2-	42	(40)	15	(14)	38	(36)	11	(10)	
ER- HER2+	35	(57)	13	(21)	12	(20)	1	(2)	
ER+ HER2-	14	(8)	20	(12)	85	(51)	49	(29)	
ER+ HER2+	56	(46)	25	(20)	37	(30)	5	(4)	
PAM50									<.001
Basal-like	56	(35)	27	(17)	54	(34)	23	(14)	
HER2-enriched	69	(63)	19	(17)	17	(15)	5	(5)	
Luminal A	2	(3)	8	(12)	40	(61)	16	(24)	
Luminal B	10	(12)	13	(15)	46	(54)	16	(19)	
Normal-like	10	(27)	6	(16)	15	(41)	66	(16)	
PAM50 grouped									<.001
Luminal	22	(12)	27	(14)	101	(54)	38	(20)	
Non-luminal	125	(46)	46	(17)	71	(26)	28	(10)	
CIT256									<.001
BasL	50	(38)	19	(15)	46	(35)	15	(12)	
mApo	45	(61)	12	(16)	13	(18)	4	(5)	
LumC	40	(43)	26	(28)	20	(22)	7	(7)	
LumB	10	(11)	12	(13)	47	(52)	22	(24)	
LumA	1	(2)	2	(5)	26	(59)	15	(34)	
NormL	1	(4)	2	(8)	20	(77)	3	(12)	
CIT256 grouped									<.001
Luminal	12	(7)	16	(10)	93	(58)	40	(25)	
Non-luminal	135	(45)	57	(19)	79	(27)	26	(9)	

P-value refer to χ^2-test or Fisher's exact test.
Abbreviations: RCB, Residual cancer burden; IHC, Immunohistochemistry; ER+, Estrogen receptor positive; ER-, ER-negative; HER2-, Human epidermal growth factor receptor-2-negative; HER2+, HER2-positive, BasL, Basal-like; mApo, molecular Apocrine; LumA, Luminal A; LumB, Luminal B; LumC, Luminal C; NormL, Normal-like, Luminal: PAM50, Luminal A/Luminal B/Normal-like; CIT256, LumA/LumB/NormL. Non-luminal: PAM50, Basal-like/HER2-enriched; CIT256, BasL/mApo/LumC.

Supplementary Table 3. Univariable logistic regression model analyses of pathological complete response according to IHC and multigene profiles.

	N	pCR	Univariate analysis		
		N (%)	Odds ratio	95%CI	*P*
IHC profile					<.001
ER-negative HER2-negative	106	42 (40)	7.22	3.69;14.1	
ER-positive HER2-negative	168	14 (8)	*Ref*		
HER2-positive	184	91 (49)	10.8	5.80;20.0	
ER-negative HER2-positive			14.8	7.02;31.2	
ER-positive HER2-positive			9.19	4.79;17.6	
PAM50					
Basal-like			4.04	1.94;8.43	<.001
HER2-enriched			12.6	5.89;27.1	
Luminal B			*Ref*		
Luminal A			0.23	0.05;1.11	
Normal-like			2.78	1.04;7.41	
PAM50 grouped					<.001
Luminal	188	22 (12)	*Ref*		
Non-luminal	270	125 (46)	6.51	3.93;10.8	
CIT256					
BasL			5.06	2.40;10.7	<.001
mApo			12.6	5.62;28.1	
LumC			6.11	2.82;13.3	
LumB			*Ref*		
LumA			0.19	0.02;1.52	
NormL			0.32	0.04;2.66	
CIT256 grouped					<.001
Luminal	161	12 (7)	*Ref*		
Non-luminal	297	135 (45)	10.3	5.51;19.4	

P-value refer to χ^2-test.
Abbreviations: pCR, Pathological complete response (Residual Cancer Burden 0); CI, Confidence interval; IHC, Immunohistochemistry; ER, Estrogen receptor; HER2, Human epidermal growth factor receptor-2, BasL, Basal-like; mApo, molecular Apocrine; LumA, Luminal A; LumB, Luminal B; LumC, Luminal C; NormL, Normal-like, Luminal: PAM50, Luminal A/Luminal B/Normal-like; CIT256, LumA/LumB/NormL. Non-luminal: PAM50, Basal-like/HER2-enriched; CIT256, BasL/mApo/LumC.

Supplementary Table 4. Multivariable logistic regression models of pathological complete response according to IHC profile and multigene profiles.

	CIT256		PAM50	
	**Odds ratio**	**95%CI**	**Odds ratio**	**95%CI**
IHC profile				
ER- HER2-	2.86	1.39;5.88	2.94	1.42;6.10
ER+ HER2-	*Ref*		*Ref*	
HER2+	6.21	3.23;12.0	7.01	3.67;13.4
Multigene profile				
Luminal	*Ref*		*Ref*	
Non-luminal	6.67	3.36;13.2	4.63	2.61;8.22

All parameters $P<.001$. $P_{heterogeneity}$=0.77 for CIT256 and 0.46 for PAM50.
Abbreviations: CI, Confidence interval; IHC, Immunohistochemistry; ER+, Estrogen receptor positive; ER-, ER-negative; HER2-, Human epidermal growth factor receptor-2-negative; HER2+, HER2-positive. Luminal: PAM50, Luminal A/Luminal B/Normal-like; CIT256, LumA/LumB/NormL. Non-luminal: PAM50, Basal-like/HER2-enriched; CIT256, BasL/mApo/LumC.

Supplementary Table 5. True classification and model predicted classification from scoring of model with IHC and CIT256 profile and IHC and PAM50 profile.

	**IHC and CIT256**		**IHC and PAM50**	
	Predicted classification		Predicted classification	
	**No pCR**	**pCR**	**No pCR**	**pCR**
**True classification**	*N*	*N*	*N*	*N*
No pCR	253	58	262	49
pCR	65	82	74	73

Abbreviations: pCR, Pathological complete response (Residual Cancer Burden 0).

Supplementary Table 6. Post-NACT surgery for patients with no *BRCA* mutation detected according to IHC biomarker and multigene profiles.

IHC profile	CIT256								PAM50							
	Luminal				Non-luminal				Luminal				Non-luminal			
	M		BCS		M		BCS		M		BCS		M		BCS	
	N	%	*N*	%	*N*	%	*N*	%	*N*	%	*N*	%	*N*	%	*N*	%
HER2-negative																
ER-negative	2	(100)	0	(0)	28	(33)	56	(67)	2	(100)	0	(0)	28	(33)	56	(67)
ER-positive	56	(52)	52	(48)	22	(49)	23	(51)	58	(50)	59	(50)	20	(56)	16	(44)
HER2-positive	20	(50)	20	(50)	61	(46)	73	(54)	28	(49)	29	(51)	53	(45)	64	(55)

Abbreviations: IHC, Immunohistochemistry; M, Mastectomy; BCS, Breast conserving surgery. ER, Estrogen receptor, HER2; Human epidermal growth factor receptor-2. Luminal: PAM50, Luminal A/Luminal B/Normal-like; CIT256, LumA/LumB/NormL, Non-luminal: PAM50, Basal-like/HER2-enriched; CIT256, BasL/mApo/LumC.

Supplementary Table 7. Axillary status before and after NACT for patients with ER-positive, HER2-negative breast cancer according to multigene profiles.

Status pre NACT	CIT256										PAM50									
	Luminal					Non-luminal					Luminal					Non-luminal				
	Status post NACT					Status post NACT					Status post NACT					Status post NACT				
	N	Neg		ITC	Pos	*N*	Neg		ITC	Pos	*N*	Neg		ITC	Pos	*N*	Neg		ITC	Pos
		N	(%)	*N*	*N*		*N*	(%)	*N*	*N*		*N*	(%)	*N*	*N*		*N*	(%)	*N*	*N*
Neg	70	31	(44)	7	32	28	22	(79)	1	5	76	35	(46)	8	33	22	18	(82)	0	4
Pos	45	**3**	**(7)**	0	42	25	**9**	**(36)**	0	16	48	**5**	**(10)**	0	43	22	**7**	**(32)**	0	15

Abbreviations: Neg, Negative; ITC, Isolated tumor cells; Pos, Positive. Luminal: PAM50, Luminal A/Luminal B/Normal-like; CIT256, LumA/LumB/NormL. Non-luminal: PAM50, Basal-like/HER2-enriched; CIT256: BasL/mApo/LumC.

Supplementary Figure 1. Forest plot from logistic regression model of pathological complete response according to IHC profile and PAM50 profile combined.

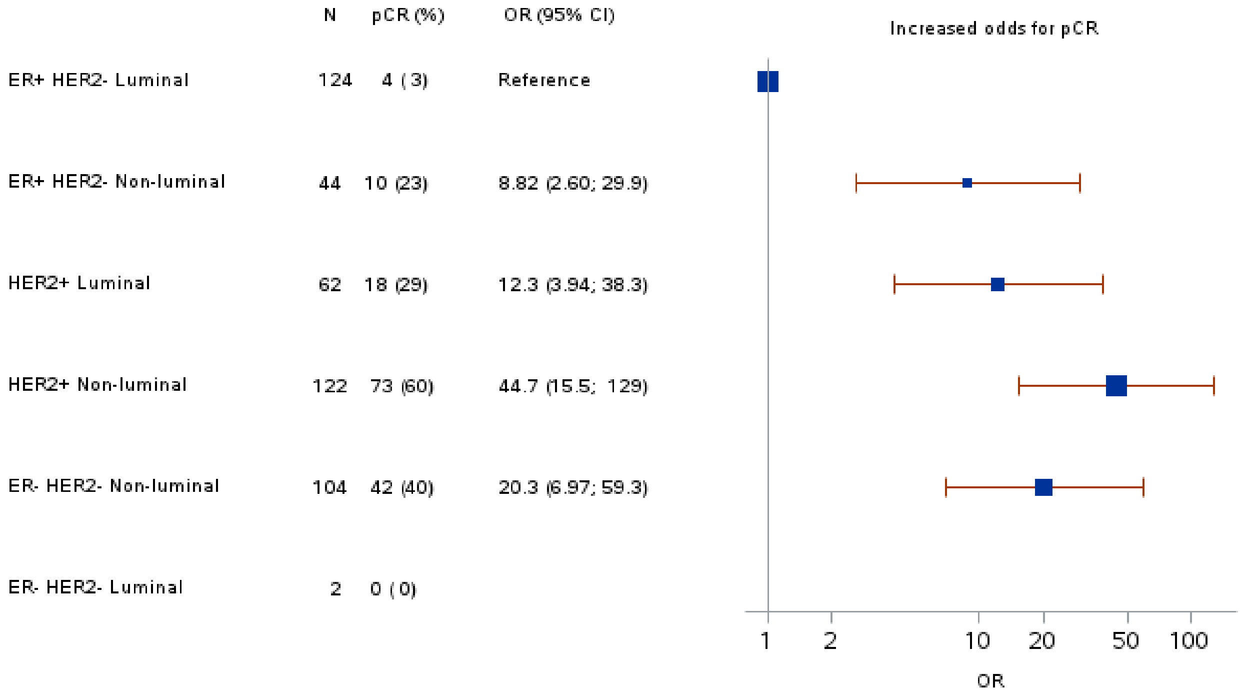

The boxes represent the weight of data in terms of sample size for each subgroup and the lines represent the 95% confidence interval of the estimated odds ratio.
Abbreviations: ER; Estrogen receptor, HER2; Human epidermal growth factor receptor-2, ER+; ER-positive, ER-; ER-negative, HER2+; Her2-positive, HER2-; HER2-negative, Luminal; Luminal A, Luminal B, Normal-like, Non-luminal; Basal-like, HER2-enriched, pCR; Pathological complete response (Residual Cancer Burden 0), OR; odds ratio.
